# Analysis of clinical diagnosis and treatment of intestinal volvulus

**DOI:** 10.1186/s12876-023-02699-2

**Published:** 2023-03-28

**Authors:** Yuhong Fo, Xiaoyu Kang, Yongqiang Tang, Lifang Zhao

**Affiliations:** 1 Xijing Hospital of Digestive Diseases, Fourth Military Medical University, Xi’an, China; 2 Department of Radiology, Xijing Hospital, Fourth Military Medical University, Xi’an, China

**Keywords:** Intestinal volvulus, Early diagnosis, Treatment, Intestinal necrosis

## Abstract

**Background:**

The aim of this study is to investigate the clinical characteristics and treatment experience of intestinal volvulus, and to analyze the incidence of adverse events and related risk factors of intestinal volvulus.

**Methods:**

Thirty patients with intestinal volvulus admitted to the Digestive Emergency Department of Xijing Hospital from January 2015 to December 2020 were selected. The clinical manifestations, laboratory tests, treatment and prognosis were retrospectively analyzed.

**Results:**

A total of 30 patients with volvulus were enrolled in this study, including 23 males (76.7%), with a median age of 52 years (33–66 years). The main clinical manifestations were abdominal pain in 30 cases (100%), nausea and vomiting in 20 cases (67.7%), cessation of exhaust and defecation in 24 cases (80%), and fever in 11 cases (36.7%). The positions of intestinal volvulus were jejunum in 11 cases (36.7%), ileum and ileocecal in 10 cases (33.3%), sigmoid colon in 9 cases (30%). All 30 patients received surgical treatment. Among the 30 patients underwent surgery, 11 patients developed intestinal necrosis. We found that the longer the disease duration (> 24 h), the higher the incidence of intestinal necrosis, and the higher the incidence of ascites, white blood cell count and neutrophil ratio in the intestinal necrosis group were significantly higher than those in the non-intestinal necrosis group (p < 0.05). After treatment, 1 patient died of septic shock after operation, and 2 patients with recurrent volvulus were followed up within 1 year. The overall cure rate was 90%, the mortality rate was 3.3%, and the recurrence rate was 6.6%.

**Conclusion:**

Laboratory examination, abdominal CT and dual-source CT are very important for the diagnosis of volvulus in patients with abdominal pain as the main symptom. Increased white blood cell count, neutrophil ratio, ascites and long course of disease are important for predicting intestinal volvulus accompanied by intestinal necrosis. Early diagnosis and timely intervention can save lives and prevent serious complications.

## Introduction

Intestinal volvulus refers to a segment of intestinal loop and its mesangial membrane twisting more than 180 degrees along the mesangial axis. Because the intestinal canal and blood supply are affected simultaneously, resulting in closed loop intestinal obstruction, which is prone to cause serious complications, such as necrosis and perforation, endangering the patient’s life [1]. Both colon and the small bowel are subject to volvulus. There are a variety of reasons that can cause a volvulus to develop including anatomically variations, medications, lifestyle, changes in physiology [2]. Some patients undergoing surgical treatment after intestinal necrosis will also develop short bowel syndrome due to excessive bowel resection, leading to poor prognosis [3]. Therefore, early diagnosis and treatment of intestinal volvulus is very important. In our study, we described the clinical data of 30 patients with intestinal volvulus in the digestive emergency department of Xijing Hospital, including clinical manifestations, laboratory examinations, imaging examinations, surgical options and prognosis, and analyzed the intestinal necrosis-related risk factors, in order to provide experience for the future clinical diagnosis and treatment of intestinal volvulus.

## Objects and methods

### Object

A total of 30 patients diagnosed with volvulus in the Digestive Emergency Department of Xijing Hospital from 2015 to 2020 were enrolled in this study.

### Data collection

The following relevant data were retrospectively collected: (1) demographic data, including age, gender, and previous surgical history; (2) Clinical symptoms, including the nature, location, degree and duration of symptoms; (3) Complete auxiliary examinations and laboratory tests after admission, including blood routine examination, ions, liver and kidney function, inflammatory factors, etc., and imaging examination including abdominal X-ray, abdominal CT and abdominal dual-source CT.

### Statistical methods

Continuous variables with normal distribution were expressed as mean ± standard deviation and compared using t-test, while continuous variables with non-normal distribution were expressed as median and interquartile range and compared using nonparametric test. For count-type data, frequency was used to describe the statistics, and Pearson chi-square test or fisher test was used to compare the differences between the two groups. Statistical significance was defined as p < 0.05.

## Results

### General information

There were 23 males (76.7%) and 7 females (23.3%), with a median age of 52 years (33–66 years), among which 9 patients (30%) were older than 60 years. The median interval between onset and admission was 23.5 h (18-38.3 h) (as shown in Table 1).

### Etiology

**Fig. 1:**
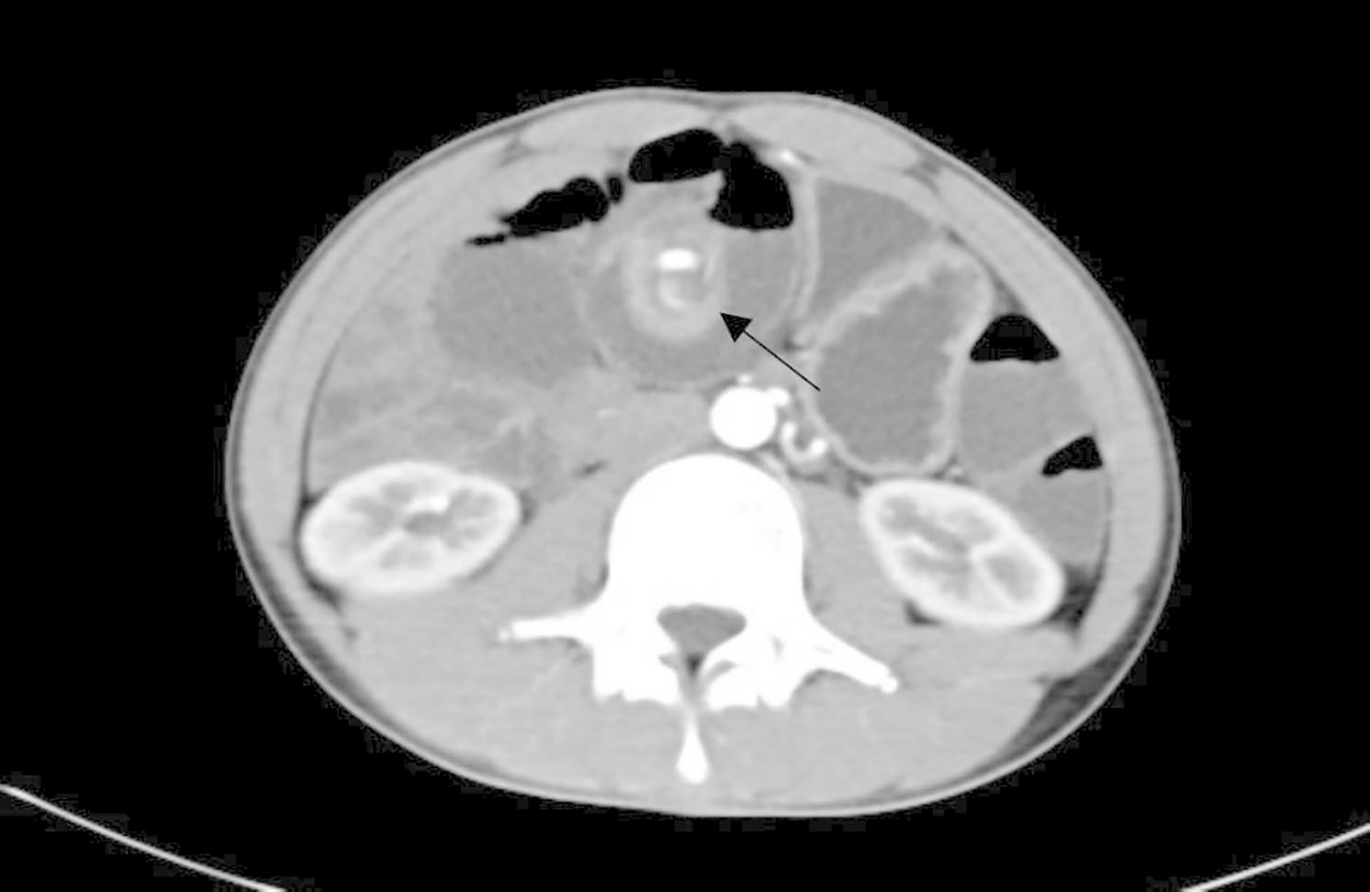
The local small intestine and connected mesentery showed swirly changes, surrounding intestinal dilatation with gas and fluid

**Fig. 2:**
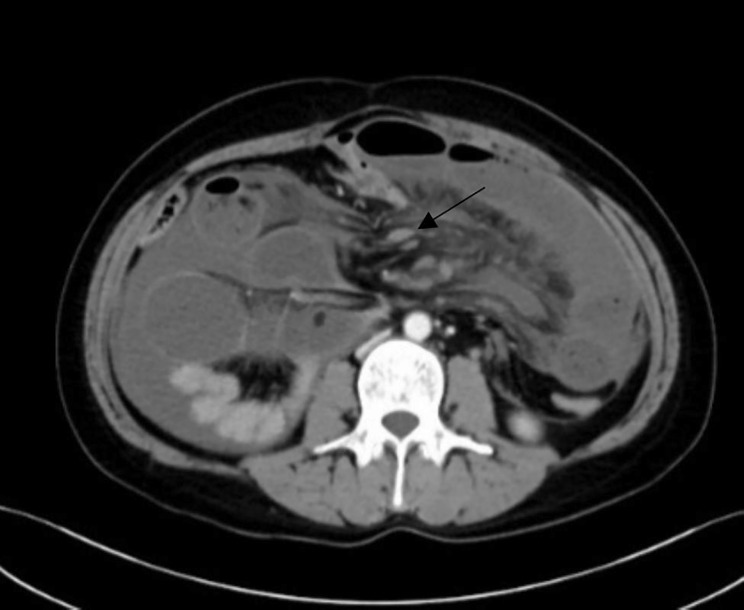
Internal abdominal hernia with small intestine volvulus

**Fig. 3:**
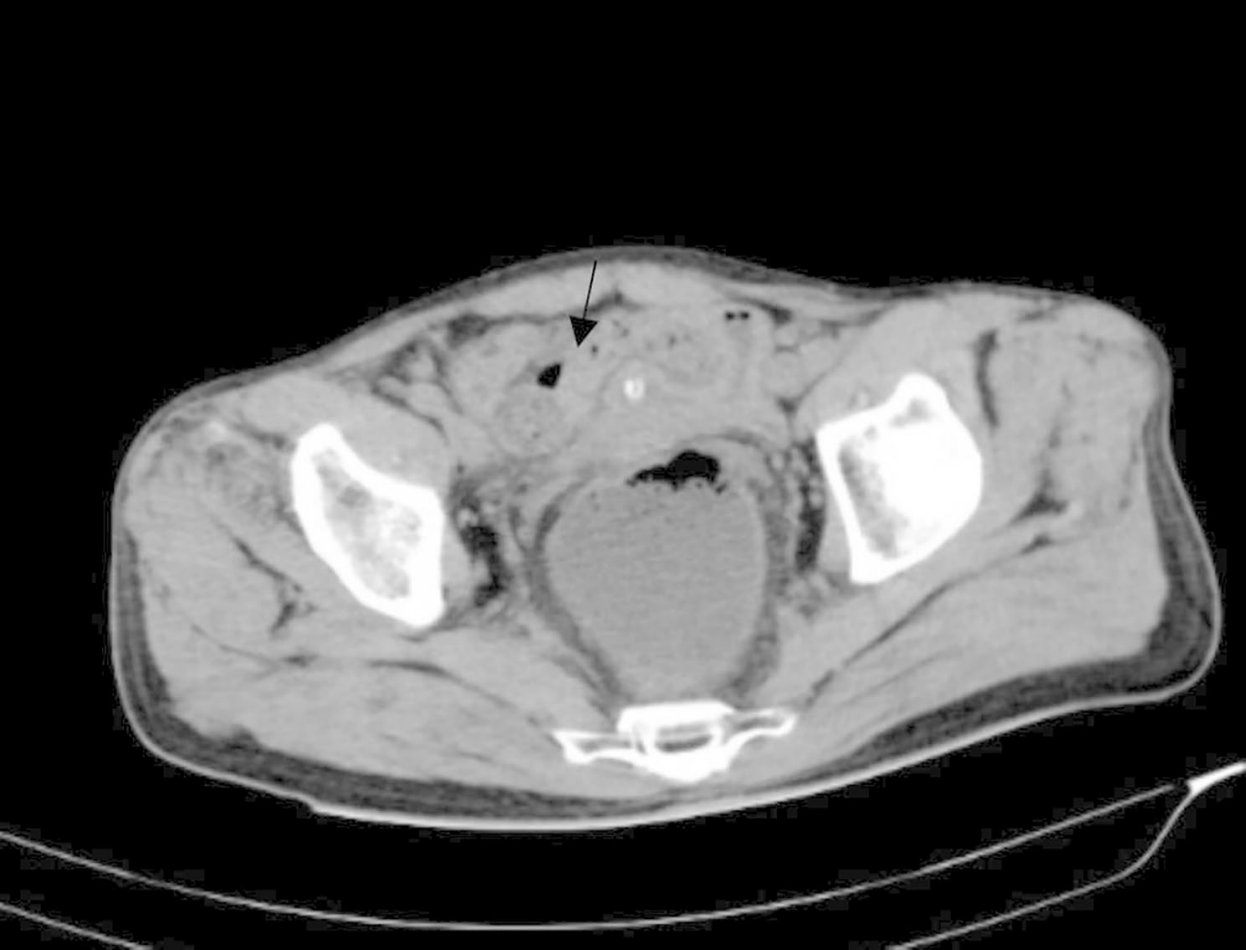
Local colon distortion with unnatural course, sigmoid colon volvulus

Among the 30 patients, there were 21 cases (70%) of small intestinal volvulus (As shown in Figs. 1 and 2), including 11 cases (36.7%) of jejunal volvulus, 10 cases (33.3%) of ileum and ileocecal volvulus, 9 patients (30%) had sigmoid volvulus (see Fig. 3). The volvulus angle ranged from 180 degrees to 1,080 degrees, and the length of necrosis varied from 30 to 70 cm in patients with intestinal necrosis (as shown in Table 1).

Most of the inducements are related to full meals and strenuous exercise after full meals, among which 17 patients had secondary intestinal volvulus, including: 2 cases of sigmoid colon cancer after operation; 1 case of abdominal closure operation due to car accident; 2 cases after caesarean operation; 1 case of total gastrectomy (esophagojejunostomy); 1 case after choledochojejunostomy; 1 case after repair of gastric perforation; 2 cases of intestinal obstruction after surgical treatment; 1 case of intestinal obstruction after conservative treatment; 1 case of jejunal stromal tumor; 2 cases of congenital malrotation of intestine; 2 cases of redundant sigmoid colon; 1 case of congenital defect of mesentery.

Eleven patients underwent partial bowel resection due to intestinal necrosis, including 4 cases with partial sigmoid colon resection and 7 cases with partial small bowel resection.

**Table 1:** General data

Clinical information	
**Age**	52 (33–66)
Gender, n (%)	
Male	23 (76.7%)
Female	7 (23.3%)
**Etiology**	
Primary	13 (43.3%)
Secondary	17 (56.7%)
**Clinical signs, n (%)**	
Abdominal pain	30 (100%)
Cessation of exhaust	24 (80%)
Nausea and vomiting	20 (66.7%)
Fever	11(36.7%)
WBC	8.7 ± 5.2
Neutrophil ratio,%	0.739 ± 0.167
**Imaging test**	
Abdominal CT Scan, n (%)	16 (69.6%)
Dual-Source CT, n (%)	12(80%)
**The site of volvulus**	
Jejunum	11 (36.7%)
Ileum and ileocecal region	10 (33.3%)
Sigmoid colon	9 (30.0%)
**Complications n (%)**	
Intestinal necrosis	11 (36.7%)
Peritonitis	26 (86.7%)

### Clinical manifestations

Abdominal pain of varying severity occurred in all the 30 patients, among which 24 patients had concomitant symptoms of absence of flatus and defecation, 20 patients had nausea and vomiting, and 11 patients had fever with the highest temperature of 38.6℃.

Signs: By physical examination, 26 patients had typical signs of peritonitis.

Laboratory examination: There were 11 patients with elevated white blood cell count (the highest was 20.7 × 109/L, with an average of 8.7 ± 5.2 × 109/L), and 14 patients with elevated neutrophil percentage (the highest was 0.946, with an average of 0.739 ± 0.167). The mean erythrocyte was 4.34 ± 0.54 × 1012/L, the mean hematocrit was 0.40 ± 0.05, the mean hemoglobin was 136.02 ± 30.47 g/L, the mean platelet was 184.27 ± 84.21/L, the mean creatinine was 85.37 ± 44.33 umol/L. The mean urea value was 6.41 ± 3.89 mm/L.

Imaging examination: Abdominal X-ray examination of 8 patients showed intestinal obstruction; Abdominal CT scan was performed in 23 patients: intestinal volvulus was found in 16 patients. Fifteen patients underwent abdominal and intestinal dual-source CT: 12 patients showed intestinal volvulus. Abdominal CT examination showed abdominal effusion in 20 patients.

### Surgical site and method

Among the 9 patients with sigmoid colon volvulus, 7 patients underwent sigmoid colon volvulus reduction, partial intestinal resection and fistulation. All these 7 patients underwent selective fistula reduction surgery, and 2 patients underwent sigmoid volvulus reduction.

Among the 6 patients with ileal volvulus, 2 patients underwent necrotic small bowel resection and ileostomy, 2 patients underwent volvulus reduction, 2 patients underwent volvulus reduction and partial bowel resection.

**Fig. 4:**
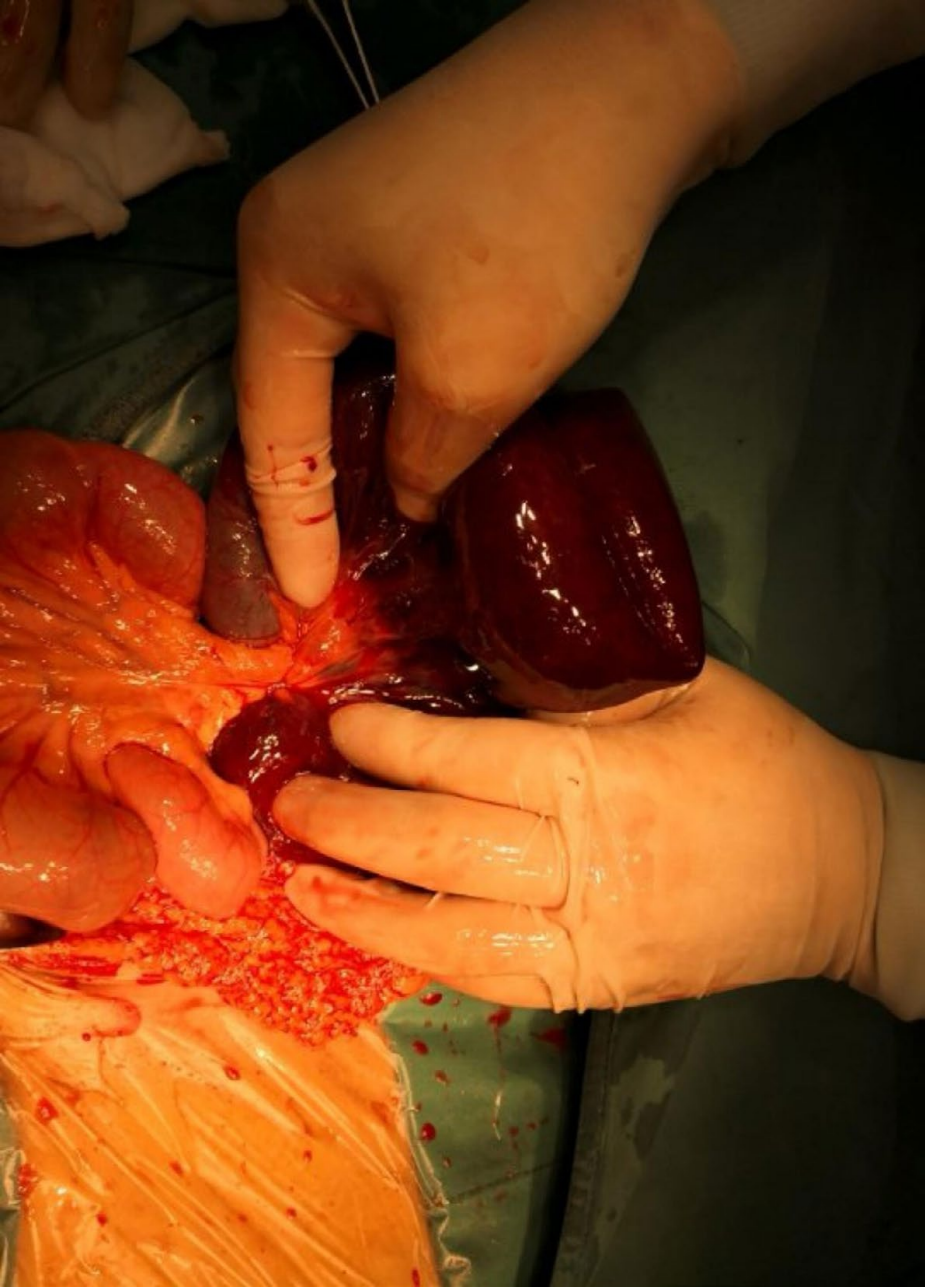
The small intestinal necrosis

Among the 4 patients with ileocecal volvulus, 1 patient underwent necrotic small bowel resection and side-to-side anastomosis of small intestine and ascending colon (as shown in Fig. 4), 2 patients underwent intestinal volvulus reduction, and 1 patient underwent tumor resection and side-to-side anastomosis of small intestine.

Of the 11 patients with jejunal volvulus, 2 patients underwent necrotic small bowel resection and side-to-side anastomosis, 1 patient underwent small bowel tumor resection (jejunal stromal tumor), and 8 patients underwent volvulus reduction (as shown in Table 2).

**Table 2:** Surgical site and method

	sigmoid colon volvulus	ileal volvulus	ileocecal volvulus	jejunal volvulus
Volvulus reduction	2	2	2	8
Volvulus reduction, partial intestinal resection and fistulation	7	4	1	2
small bowel tumor resection			1	1
Total	9	6	4	11

The operation showed a moderate amount of bleeding ascites in the abdominal cavity, and no malodor was heard. The adhesion band on the ileal surface 1 m from the ileocecal region wrapped around the ileocecal small intestine and the intestinal tube 50 cm above the ileocecal region, forming a constriction ring. The small intestine 50 cm above the ileocecal region was black and dull, and the peristalsis disappeared. The necrotic small intestine was removed by side-to-side anastomosis of proximal small intestine and ascending colon, using an Endovascular gastrointestinal anastomosis stapler (ENDO-GIA).

### Complications and prognosis

Of the 30 patients, 11 underwent partial bowel resection for intestinal necrosis. After statistical analysis, the occurrence of intestinal necrosis was not related to whether it was secondary or whether there were signs of peritonitis, but the duration of disease was longer, the incidence of ascites was higher, and the preoperative white blood cell count and neutrophil ratio were significantly increased in the intestinal necrosis group (all p < 0.05) (as shown in Table 3).

After operation, a 92-year-old patient with jejunal volvulus died of septic shock. One patient with sigmoid colon volvulus developed pulmonary infection after operation and was discharged after 1 week of intensive care unit treatment. A 69-year-old patient with ileal volvulus developed anemia after surgery and was discharged after symptomatic treatment. One patient with small intestinal volvulus had wound dehiscence due to strenuous activity 2 weeks after operation, and the skin grafts were placed under the incision for incision decompression suture.

Postoperative follow-up: 29 patients were followed up for 1 year, and 2 patients underwent second surgery due to volvulus. The overall cure rate was 90%, the mortality rate was 3.3% and the recurrence rate was 6.6%.

**Table 3:** Subgroup analysis of etiological intestinal necrosis

	Intestinal volvulus with intestinal necrosis (n = 11)	Intestinal volvulus without intestinal necrosis (n = 19)	P (Fisher’s exact test probabilities)
**Pattern**			p = 0.259
Primary	3	10	
Secondary	8	9	
**Disease course**			p < 0.05
Less than 24 h	4	15	
More than 24 h	7	4	
**Signs**			p = 1
Signs of peritonitis	10	16	
Signs of non-peritonitis	1	3	
**Ascites**			p < 0.05
Ascites	10	10	
Without Ascites	1	9	
**Laboratory inspection**			
WBC(*10^9^)	11.28 ± 6.23	6.90 ± 3.54	p < 0.05
NEUT (%)	0.83 ± 0.11	0.69 ± 0.18	p < 0.05

## Discussion

The incidence of intestinal volvulus is high in Asia, with 24–60 cases per 100,000 people [4]. The causes are mainly related to anatomical factors such as adhesion, intestinal diseases, and physical factors such as tumors and ascariasis. No matter where it occurs, it has the characteristics of acute onset, severe condition and rapid development, which is easy to cause intestinal blood circulation disorders, and can lead to intestinal ischemic necrosis, perforation, and even septic shock and death in the early stage [5]. Therefore, early diagnosis and timely treatment are very important.

Abdominal pain is one of the most common and typical clinical manifestations of intestinal volvulus. Abdominal pain is generally manifested as sudden and persistent abdominal pain, which is colic in nature and can radiate to the lower back, accompanied by nausea, vomiting, cessation of exhaust, cessation of defecation, ascites, etc. [6]. In this study, it was found that all patients had abdominal pain of different degrees, and 63.3% of them were accompanied by symptoms of anal exhaust cessation. Abdominal distension generally occurred in the late stage, which may be caused by the expansion of the intestinal tube above the site of intestinal obstruction. When the signs of peritonitis appear, it often indicates that there are adverse complications. In this study, 26 patients were found to have signs of peritonitis, but statistical analysis showed that there was no significant relationship between intestinal necrosis and signs of peritonitis, which may be related to the small sample size.

Previous studies have shown that small intestinal volvulus can occur at any age, while sigmoid colon volvulus is more common in elderly men, mainly due to the long sigmoid colon and relatively short mesangial vessels, or caused by inflammation and adhesion, which is related to long-term constipation [7]. We found that elderly patients accounted for 44.4% of the 9 patients with sigmoid volvulus. Acute small intestinal torsion is more common in young adults. In this study, among 21 patients with small intestinal volvulus, young and middle-aged patients accounted for 76.2%. Considering that this study is a small sample study, large sample studies are needed to confirm the incidence of volvulus in all ages.

Early imaging examination after admission can clarify the diagnosis of volvulus. Orthostatic abdominal plain film is an important imaging method for the initial screening of suspected intestinal obstruction. Patients with intestinal obstruction generally present with large isolated intestinal loops with general intestinal distensions or obvious inflation and stair-steping multiple air-fluid levels. In this group, 15 patients underwent abdominal upright plain radiography examination, and 8 patients showed intestinal obstruction. This indicates that for patients with abdominal pain and suspected intestinal obstruction, abdominal upright plain film has a high diagnostic value for intestinal obstruction. In the case of highly suspected volvulus based on clinical symptoms and imaging examinations, plain abdominal scan or enhanced CT is an essential imaging method for the definite diagnosis of volvulus. The obvious “sign of whirlpool, sign of beak, and target loop” found in abdominal CT can be considered for the diagnosis of volvulus [8–10]. We found that the positive diagnosis rate of abdominal CT plain scan for volvulus was 69.6% (16/23). In addition, due to the characteristics of “high resolution”, the accuracy rate of intestinal dual-source CT for the diagnosis of intestinal volvulus can reach 80%. It can not only find the morphological changes of the lesions, but also determine the location, range, degree of the lesions and the causes of some diseases. In our study, the diagnostic rate of intestinal dual-source CT for volvulus was 80%, which was similar to the rate reported in previous literature [9]. This study shows that orthostatic abdominal plain radiography for patients with abdominal pain is beneficial for early screening of patients with volvulus, and CT examination is preferred for patients with highly suspected volvulus in clinic.

The treatment of volvulus includes non-surgical treatment, endoscopic treatment and surgical treatment. Non-surgical treatment includes symptomatic and supportive treatment such as gastrointestinal decompression, fluid replacement, anti-infection, and correction of electrolyte acid-base balance disorder. It is suitable for mild conditions without severe symptoms such as intestinal ischemia. Once the disease progresses or becomes worse, emergency surgery should be performed immediately [11]. Laparoscopic colorectal surgery has been the subject of discussions in terms of indications and results. Previous studies find that the use of laparoscopy for the management of sigmoid volvulus is safe, feasible and associated with a low prevalence of complications (operative duration, infection, blood loss, hospital stay, recurrence, conversion, morbidity, mortality). Laparoscopy was used for different situations to treat sigmoid volvulus. The most frequent indication was uncomplicated sigmoid volvulus after endoscopic detorsion (96.5%) [12]. Since our hospital has not carried out colonoscopy examination and reduction of sigmoid colon torsion, colorectal resection are still performed open. In our study, the condition of the 30 patients did not improve significantly under supportive treatment, all the 30 patients underwent surgical treatment. In surgical treatment, the small intestinal volvulus should be treated as soon as possible. During surgical exploration, the intestinal tube should be decompress and reduced, and then the blood supply of the intestinal canal and mesentery should be observed. If there is a necrotic intestinal tube, the necrotic intestinal segment should be resected to prevent abdominal infection caused by endotoxin in the intestinal cavity. When removing the intestinal tube, it should be reasonably selected according to the necrotic tissue and local intestinal blood supply, and it should not be too long to avoid the occurrence of postoperative short bowel syndrome. Generally, the resection range should be 3 ~ 5 cm beyond the necrotic site [13]. In this study, among the 17 patients with intestinal volvulus, 11 patients without intestinal necrosis underwent surgical reduction, while 6 patients with intestinal necrosis underwent resection of necrotic bowel. For volvulus of the sigmoid colon, if it is a congenital anatomical abnormality, it can be treated with reduction and fixation without resection after evaluation. Simple reduction of sigmoid colon has a high recurrence rate of 50%. Intraoperative reduction and the second-stage sigmoid colon resection are the most effective methods. In this group of 9 patients with sigmoid colon torsion, 7 patients with sigmoid colon volvulus underwent partial intestinal resection with volvulus reduction of sigmoid colon and fistulation, and 7 patients underwent ostomy reentry operation. 2 patients underwent sigmoid volvulus reduction.

Intestinal necrosis is one of the serious complications after intestinal volvulus [14]. In this study, 11 of the 30 patients had intestinal necrosis with an incidence of 36.7%. The length of intestinal necrosis ranged from 30 to 70 cm. Intestinal necrosis must be excised. If not, it will cause more serious complications such as peritonitis, infection, toxic shock and so on. However, excessive resection of necrotic bowel can also cause short bowel syndrome and reduce the quality of life of patients. Therefore, early identification and prevention of intestinal necrosis is also very important. Through statistical analysis, we found that the occurrence of intestinal necrosis and the cause of intestinal torsion were not significantly related to the presence or absence of signs of peritonitis, while the longer course of disease (> 24 h), massive ascites, and the significant increase in white blood cell count and neutrophil percentage were often accompanied by the occurrence of intestinal necrosis. We think because of the small sample size, conclusions about peritonitis are not necessarily accurate. Therefore, in clinical practice, we should be alert to the above clinical manifestations and laboratory test results of patients with volvulus, and perform surgery as soon as possible to avoid the occurrence of intestinal necrosis and improve the prognosis of patients.

## Conclusion

Intestinal volvulus is a relatively rare disease, and its clinical manifestations are similar to other acute abdominal diseases. The lack of specific symptoms and signs in the early stage often leads to misdiagnosis or missed diagnosis, resulting in serious complications. In clinical diagnosis and treatment, it is necessary to combine the patient’s clinical manifestations, dynamically monitor the patient’s abdominal pain symptoms and signs, and combine laboratory tests and imaging examinations to make an early diagnosis. In this study, it was also found that the significant increase in the ratio of white blood cells to neutrophils, the appearance of ascites, and the prolonged disease course in patients with volvulus often suggested an increased incidence of intestinal necrosis.

## Data Availability

The data that support the findings of this study are available on request from the corresponding author.
